# Flecainide Provides Comparable Rhythm Control Efficacy to Repeat Radiofrequency Ablation in Patients with Paroxysmal Atrial Fibrillation After Failed Cryoballoon Ablation

**DOI:** 10.19102/icrm.2026.17035

**Published:** 2026-03-15

**Authors:** Mustafa Lütfullah Ardıç, Halil Coskun, Hilmi Erdem Sumbul, Yahya Kemal Icen, Abdullah Eren Çetin, Mevlut Koç

**Affiliations:** 1 Department of Cardiology, University of Health Sciences, Adana Health Practice and Research Center, Adana, Turkey; 2 Department of Internal Medicine, University of Health Sciences, Adana Health Practice and Research Center, Adana, Turkey; 3 Department of Cardiology, 25 Aralık State Hospital, Gaziantep, Turkey

**Keywords:** Ablation, atrial fibrillation, flecainide, recurrence

## Abstract

Class IC anti-arrhythmic drugs are primarily preferred for the rhythm control of atrial fibrillation (AF). In cases where ablation fails, the appropriate rhythm-control strategy is still unclear. In our study, we aimed to evaluate the efficacy of flecainide, propafenone, and radiofrequency ablation (RFA) as rhythm-control strategies in paroxysmal AF patients with failed cryoballoon ablation (CBA). In this cross-sectional study, 1120 patients who underwent CBA for paroxysmal AF between 2017 and 2024 were screened. Within this patient group, 230 patients with recurrent AF (≥3 months) after CBA were identified. A total of 120 patients (40 cases per treatment) who underwent rhythm control and received flecainide, propafenone, or RFA were finally included in the study. Study participants were then divided into three groups, receiving flecainide (group I), propafenone (group II), or RFA (group III). All patients were followed up for at least 1 year for AF recurrence, which was confirmed in 52 (43.2%) patients. The AF recurrence rates in groups I, II, and III were 35%, 75%, and 20%, respectively. Although the frequency of AF recurrence in groups I and III was statistically similar (*P* > .05), it was significantly lower in these groups than that in group II (*P* < .05). Group I patients were significantly more likely to use β-blockers than group II or III patients (*P* < .05). Patients with AF recurrence had a larger left atrial (LA) diameter and greater propafenone use. The number of patients who used flecainide and underwent RFA was lower in the AF recurrence group. In logistic regression analysis, LA diameter was found to be an independent predictor of AF recurrence (*P* = .002). In conclusion, based on the findings of our study, flecainide therapy can be used with an acceptable success rate in patients with recurrent AF after CBA.

## Introduction

Atrial fibrillation (AF) has a prevalence of approximately 2%–3% in the general population and is the most common supraventricular arrhythmia.^1^ According to the European Society of Cardiology (ESC) guidelines on the diagnosis and management of AF, in patients with paroxysmal AF without structural heart disease, class IC anti-arrhythmic drugs (AADs) and AF ablation are recommended with class I and class IIa indications, respectively.^1^ Furthermore, the same guidelines recommend radiofrequency ablation (RFA) or cryoballoon ablation (CBA) with a class I indication for maintaining sinus rhythm in symptomatic patients with paroxysmal or persistent AF who have failed at least one AAD.^1^

Unfortunately, the outcomes of both AAD therapy and ablation procedures remain suboptimal. Post-ablation AF recurrence has been reported in approximately 20%–37% of cases.^1–6^ Flecainide, encainide, and propafenone are class Ic AADs. Their primary mechanism of action involves potent inhibition of the Nav1.5 sodium channel, which is responsible for the rapid inward sodium current during phase 0 of the cardiac action potential, resulting in fast depolarization of myocardial cells. The ESC guidelines recommend class Ic agents as part of a long-term rhythm-control strategy in AF patients without structural heart disease.^1^

However, treatment strategies and algorithms for AF recurrence following ablation are not clearly defined, either in the current ESC guidelines or in clinical practice. To the best of our knowledge, there is insufficient evidence regarding the efficacy of flecainide or propafenone in patients who experience recurrent AF following CBA. In clinical practice, these patients are often managed as if they are presenting with paroxysmal AF for the first time. Treatment choices between AADs and ablation are made using a multidisciplinary approach involving the patient, family members, physicians, and nursing staff.

In this study, we aimed to evaluate the efficacy of flecainide, propafenone, and RFA in rhythm control among patients with recurrent paroxysmal AF after undergoing CBA.

## Materials and methods

### Study population

For our study, patients who underwent CBA for symptomatic paroxysmal AF in the electrophysiology laboratory of the Cardiology Department at our hospital between July 1, 2017, and May 1, 2024, and subsequently experienced AF recurrence during follow-up (≥3 months) were retrospectively screened. Among the 230 patients identified with AF recurrence, after applying the exclusion criteria, 120 patients were enrolled and divided into three groups, each consisting of 40 patients: group I included those treated with flecainide, group II included those treated with propafenone, and group III included those treated with RFA. The study cohort consisted of 70 men and 50 women, with a mean age of 59.5 ± 14.2 years. The diagnosis and treatment of AF were carried out according to the most recent ESC guidelines.^1^

Patients with persistent AF; those who experienced adverse events or side effects leading to the discontinuation of flecainide or propafenone; those who had complications or unsuccessful procedures during RFA; those with acute or end-stage liver or kidney disease, ischemic heart disease, end-stage chronic obstructive pulmonary disease, malignancy, and/or active infection within the last 2 weeks; those with bleeding disorders, a history of hemorrhagic stroke, severe aortic or mitral valve disease, or congestive heart failure; those with a left atrial (LA) diameter of >55 mm, left ventricular (LV) systolic dysfunction (LV ejection fraction [LVEF] <50%), or a life expectancy of <1 year; and those who did not provide informed consent were excluded.

All participants were fully informed about the study, and written informed consent was obtained. The necessary ethical approval was obtained from the ethics committee of the Health Sciences University Adana City Training and Research Hospital (decision no. 505).

Each patient underwent a detailed medical history-taking and comprehensive physical examination. Baseline resting heart rate in sinus rhythm was recorded. Information was collected regarding coexisting cardiac and non-cardiac systemic diseases, thromboembolic risk factors, and any anticoagulant medications being used. The CHA₂DS₂-VASc score was calculated based on the parameters outlined in the 2024 ESC AF guidelines.^1^

All patients underwent laboratory evaluation, including serum biochemistry and complete blood count. Biochemical tests included measurements of urea, creatinine, uric acid, total cholesterol, triglycerides, low-density lipoprotein cholesterol, high-density lipoprotein cholesterol, and C-reactive protein. White blood cell counts were also obtained as part of the complete blood count.

All patients underwent M-mode and two-dimensional transthoracic echocardiography in the echocardiography laboratory prior to the procedure. Measurements were performed using an EPIC 7C echocardiography system (Philips Healthcare, Andover, MA, USA). LV diameters were measured in the parasternal long-axis view with the M-mode cursor placed just beyond the tips of the mitral valve leaflets and perpendicular to the LV long axis. LVEF was calculated using the Simpson method from apical two-chamber and four-chamber views. The LA diameter was measured at end-diastole in the parasternal long-axis view.^7^

### Initiation of anti-arrhythmic therapy

Before initiating AAD therapy, all patients in AF rhythm underwent electrical and/or pharmacological cardioversion to restore sinus rhythm. For patients already in sinus rhythm at baseline, treatment was initiated directly. Prior to the administration of flecainide or propafenone, each patient was evaluated for contraindications or conditions that would make the use of either drug inappropriate.

Flecainide was initiated at a dose of 100 mg twice daily, in accordance with the current recommendations. Propafenone was administered at a dose of 150 mg three times daily. All patients were monitored for adverse effects and proarrhythmic events associated with the medications at 1-month and 3-month intervals during follow-up.

### Electrophysiological study and radiofrequency ablation method

The RFA procedures were performed by seven experienced electrophysiologists, each of whom had performed at least 100 AF ablation procedures annually for a minimum of 5 years. The procedures were carried out in teams of two operators. All patients underwent a comprehensive pre-procedural evaluation by an anesthesiologist.

Prior to the procedure, all patients underwent transesophageal echocardiography under anesthesia to assess the LA appendage. Eligible patients were then transferred to the electrophysiology laboratory for RFA. The procedures were initiated with the administration of midazolam and fentanyl under the supervision of the anesthesia team. Local anesthesia was applied to the right and left femoral regions. Venous access was obtained via a single puncture in each femoral vein. A decapolar catheter was inserted into the coronary sinus via a 6-Fr venous sheath through the left femoral vein (Johnson & Johnson MedTech, New Brunswick, NJ, USA). From the right femoral vein, an 8-Fr catheter was introduced and exchanged for a long sheath (PREFACE Guiding Sheath; Johnson & Johnson MedTech) for transseptal puncture. LA access was obtained using a transseptal needle (HeartSpan^®^; Merit Medical, Malvern, PA, USA). An 8-Fr deflectable sheath (Agilis™ steerable introducer; Abbott, Chicago, IL, USA) was then placed. LA mapping was performed using electroanatomic mapping systems: CARTO^®^ 3 (Biosense Webster, Diamond Bar, CA, USA) and EnSite™ X (Abbott), with the assistance of PentaRay™ (D128211; Biosense Webster) and HD Grid (Advisor FL™ Sensor Enabled; Abbott) catheters. Pulmonary vein (PV) potentials in sinus rhythm were assessed for entrance and exit block. PVs were classified as isolated or non-isolated using WorkMate™ Claris™ (Abbott). RFA of the PV antral regions was performed using either the Thermocool^®^ Smarttouch™ SF/Thermocool^®^ SF irrigated catheter (Biosense Webster) or the TactiCath™ irrigated catheter (Abbott). The ablation energy settings were 25–30 W for the LA roof and posterior wall and 35–40 W for other regions. In cases where no active PV triggers were identified, isoproterenol was administered to unmask potential AF foci. Identified foci were ablated using RF energy. PV isolation was re-assessed 20 min post-ablation to confirm durability. In patients with documented or inducible atrial flutter, macro–re-entrant tachycardias were mapped, and RF ablation lines were applied to the “early meets late” zones to terminate the arrhythmia and restore sinus rhythm. Following the procedure, all patients were transferred to the post-anesthesia care unit for stabilization before being moved to their respective hospital wards.

### Clinical follow-up and recurrence detection

Following the restoration of sinus rhythm in all patients, follow-up visits were scheduled at 1, 3, 6, 9, and 12 months. At each visit, patients were evaluated for potential drug-related side effects and AF recurrence. These assessments included a detailed clinical history, comprehensive physical examination, and routine 12-lead electrocardiography (ECG). Additionally, each patient underwent 72-h ambulatory rhythm Holter monitoring, performed no earlier than the third month after study enrollment. Patients who experienced AF-related symptoms such as palpitations between scheduled visits were instructed to contact the clinic. These symptomatic patients were then evaluated for recurrence using both 12-lead ECG and rhythm Holter recordings. AF recurrence was defined as any episode of continuous AF lasting >30 s as documented on 12-lead ECG or rhythm Holter monitoring. In patients who underwent RFA, AF episodes occurring within the first 3 months after the procedure were not classified as recurrences. Patients with no documented AF episodes during follow-up were classified as the non-recurrence group, whereas those with confirmed AF episodes were classified as the recurrence group.

### Statistical analysis

Variables were classified as either categorical or continuous. Categorical variables were presented as frequencies and percentages, while continuous variables were expressed using mean ± standard deviation (SD) values. The normality of the distribution of continuous variables was assessed using the Shapiro–Wilk test. Additionally, the Kolmogorov–Smirnov test was also used to evaluate whether continuous variables followed a normal distribution. Continuous variables were reported with mean ± SD values, and categorical variables were expressed as numbers and percentages. Comparisons of continuous variables among the three groups were performed using one-way analysis of variance (ANOVA) for normally distributed data and the Kruskal–Wallis one-way ANOVA for non-normally distributed data. For post hoc comparisons of normally distributed variables, the Scheffé or Games–Howell tests were used depending on the homogeneity of variances. For non-normally distributed variables, Bonferroni-adjusted Mann–Whitney *U* tests were employed for multiple comparisons. To assess the differences in categorical variables, Fisher’s exact test was used. Comparisons between two groups for continuous variables were performed using Student’s *t* test for normally distributed data and the Mann–Whitney *U* test for non-normally distributed data. The chi-squared test was used to compare categorical variables. To identify independent predictors of AF recurrence, all parameters found to be statistically significant (*P* < .05) in the univariate analysis were subsequently included in a multivariate logistic regression model. All statistical analyses were performed using IBM SPSS Statistics version 24.0 (SPSS Inc., Chicago, IL, USA), and *P* < .05 was considered statistically significant.

## Results

Patients with recurrent paroxysmal AF included in the study were categorized into three groups according to the rhythm-control strategy applied, as previously described: group I (flecainide), group II (propafenone), and group III (RFA). All clinical and demographic parameters of the enrolled patients were compared across these three groups. The study population was followed up for a mean duration of 476 ± 66 days. At the end of the follow-up period, AF recurrence was detected in 52 patients (43.2%). Parameters associated with AF recurrence were identified and analyzed. Among the 40 patients with AF recurrence who underwent CBA, PV reconnection was identified in 38 patients (95%). A total of 47 PVs demonstrated reconnection, including nine patients with two PVs and 29 patients with a single PV reconnection, respectively. The distribution of reconnected veins was as follows: left superior PV in nine cases, left inferior PV in 14 cases, right superior PV in 16 cases, and right inferior PV in eight cases.

### Demographic, clinical, medical treatment, and laboratory data of the patient groups

The demographic, clinical, and medical treatment characteristics of the patient groups are presented in **Table 1**. Upon evaluation of the demographic and clinical parameters across all groups, no significant differences were observed, indicating that the groups were comparable in terms of baseline characteristics. However, it was noted that all patients in group I were receiving β-blocker therapy, and the rate of β-blocker use was significantly greater in this group compared to both group II and group III (*P* < .05). Furthermore, metoprolol was the exclusive β-blocker used in group I. The laboratory findings of the study population are summarized in **Table 2**. Evaluation of laboratory parameters revealed no statistically significant differences among the three groups, suggesting that the groups were similar in terms of biochemical and hematological profiles.

**Table 1:** Demographic, Clinical, and Medical Treatment Findings of the Study Groups

Variable	Group I (n = 40)	Group II (n = 40)	Group III (n = 40)	***P*** Value
Age, years	61.5 ± 12.4	58.9 ± 13.1	56.8 ± 12.5	.178
Gender (female), n	20 (50)	14 (35)	16 (40)	.339
Heart failure, n (%)	4 (10)	4 (10)	2 (5)	.404
Hypertension, n (%)	16 (40)	18 (45)	20 (50)	.645
Diabetes mellitus, n (%)	7 (18)	6 (15)	8 (20)	.467
Coronary artery disease history, n (%)	6 (15)	6 (15)	10 (25)	.256
Previous cerebrovascular accident, n (%)	5 (13)	4 (10)	4 (10)	.545
Systolic blood pressure, mmHg	129 ± 12	128 ± 13	124 ± 13	.145
Diastolic blood pressure, mmHg	80 ± 7.8	81 ± 9.8	79 ± 9.5	.285
Heart rate, beats/min	74 ± 10	78 ± 13	72 ± 16	.165
CHA_2_DS_2_-VASc score	1.6 ± 1.5	1.4 ± 1.6	1.4 ± 1.7	.345
ACEI or ARB use, n (%)	20 (50)	16 (40)	18 (45)	.374
β-Blocker use, n (%)	40 (100)^a,b^	32 (80)	28 (70)	**.001**
Oral anticoagulant use, n (%)	36 (90)	36 (90)	32 (80)	.378
Maintenance sinus rhythm ≥1 year, n (%)	26 (65)^a^	10 (25)^c^	32 (80)	**.032**

*Abbreviations:* 3D, three-dimensional; ACEI, angiotensin-converting enzyme inhibitor; ARB, angiotensin receptor blocker. Values are shown as mean ± standard deviation or n (%). Group I = flecainide group, group II = propafenone group, and group III = 3D radiofrequency catheter ablation group. ^a^Significant association between groups I and II (*P* < .05). ^b^Significant association between groups I and III (*P* < .05). ^c^Significant association between groups II and III (*P* < .05). Statistically significant *P* values are shown in bold

**Table 2:** Laboratory Findings of the Study Groups

Variable	Group I (n = 40)	Group II (n = 40)	Group III (n = 40)	***P*** Value
White blood cells, μL	8.5 ± 2.2	7.4 ± 1.6	8.2 ± 2.9	.172
Hemoglobin, g/dL	13.2 ± 1.8	13.9 ± 2.7	13.3 ± 1.7	.467
Platelet count, 10^3^/μL	248 ± 69	229 ± 76	264 ± 60	.138
Blood urea nitrogen, mg/dL	32 ± 8.1	29 ± 7.2	34 ± 9.2	.146
Creatinine, mg/dL	0.86 ± 0.27	0.81 ± 0.14	0.82 ± 0.23	.178
Total cholesterol, mg/dL	181 ± 44	183 ± 43	193 ± 54	.511
Low-density lipoprotein cholesterol, mg/dL	122 ± 25	116 ± 26	125 ± 38	.481
High-density lipoprotein cholesterol, mg/dL	47 ± 15	46 ± 12	45 ± 13	.752
Triglyceride, mg/dL	140 ± 52	192 ± 78	182 ± 88	.183
hs-CRP, mg/L	0.41 ± 0.10	0.43 ± 0.34	0.47 ± 0.38	.566
LVEF, %	57 ± 4.6	56 ± 6.8	58 ± 4.3	.676
Left atrial dimension, mm	43 ± 3.4	43 ± 2.8	41 ± 5.9	.072

*Abbreviations:* 3D, three-dimensional; hs-CRP, high-sensitivity C-reactive protein; LVEF, left ventricular ejection fraction. Values are shown as mean ± standard deviation. Group I = flecainide group, group II = propafenone group, and group III = 3D radiofrequency catheter ablation group.

### Treatment efficacy

The overall efficacy of class Ic AADs in maintaining sinus rhythm for ≥1 year was found to be 45%. When assessed separately, the maintenance of sinus rhythm for ≥1 year was achieved in 65% of patients receiving flecainide and 25% of those receiving propafenone **(Table 1)**. This difference between flecainide and propafenone was statistically significant (*P* < .05), indicating the superior efficacy of flecainide in long-term rhythm control. The RFA group demonstrated maintenance of sinus rhythm for ≥1 year in 80% of patients. When compared with flecainide, the efficacy of RFA was found to be statistically similar (*P* > .05), indicating comparable long-term rhythm-control outcomes between these two interventions. However, RFA was significantly more effective than propafenone in maintaining sinus rhythm for ≥1 year (*P* < .05), highlighting the inferiority of propafenone in this regard.

### Parameters associated with atrial fibrillation recurrence

The patients included in the study were divided into two groups (52 and 68 patients, respectively) with and without AF recurrence after at least 1 year of follow-up. Demographic, clinical, medical treatment, and laboratory data were compared between the groups with and without AF recurrence. The parameters found to be different between the two groups are shown in **Table 3**. Patients with AF recurrence had a larger LA diameter, and a higher number of patients were on propafenone. The number of patients who used flecainide and underwent RFA was lower in the AF recurrence group. Multivariate logistic regression analysis was performed to identify the parameters that were closely and independently associated with AF recurrence in the patients included in the study. As a result, LA diameter was found to be an independent predictor of AF recurrence (*P* = .002). Each 1-mm increase in the LA diameter predicted AF recurrence at a rate of 21.3%.

**Table 3:** Parameters Found to be Different in Patients with and Without AF Recurrence

Variable	With AF Recurrence (n = 68)	Without AF Recurrence (n = 52)	***P*** Value
Left atrial dimension, mm	42 ± 4.5	45 ± 4.5	**<.001**
Flecainide use, n (%)	26 (38)	14 (27)	**.034**
Propafenone use, n (%)	10 (15)	30 (58)	**.001**
Radiofrequency ablation, n (%)	32 (47)	8 (15)	**<.001**

*Abbreviation:* AF, atrial fibrillation. Statistically significant *P* values are shown in bold.

## Discussion

Our study yielded several important findings. (1) the maintenance of sinus rhythm for ≥1 year was achieved in 65% of patients treated with flecainide and 25% of those treated with propafenone. Flecainide was significantly more effective than propafenone in achieving sustained rhythm control over 1 year. (2) The efficacy of RFA in maintaining sinus rhythm for ≥1 year was found to be 80%. Although RFA was generally more effective than class IC AADs, its efficacy was statistically comparable to that of flecainide. To the best of our knowledge, this is the first study to evaluate the efficacy of class IC AADs in patients with recurrent AF following CBA. (3) In line with previous studies, LA diameter was identified as an independent predictor of AF recurrence in our cohort, even in patients undergoing a second-line rhythm-control strategy after CBA.

The management of AF includes two major components: rhythm or rate control with AADs and thromboembolic prophylaxis.^1^ The primary goal of rhythm control is to restore and maintain sinus rhythm. CBA has become a prominent method in rhythm-control strategies, with RFA serving as an effective alternative. The primary target in AF ablation is PV isolation, which can be effectively achieved with CBA.^1^ In a meta-analysis of 19 clinical trials, the recurrence rate of AF after CBA over a 3-year follow-up period was reported to be approximately 37%.^2^ Similarly, several studies from our center have shown post-CBA AF recurrence rates of 20%–22%.^3–6^ Key predictors of recurrence after CBA include LA size, persistent AF, early recurrence, and AF duration. Among these, LA diameter has been recognized as the most significant predictor of recurrence in patients undergoing rhythm control via either ablation or AAD therapy.^1,5,8^ In our study, LA size remained the most influential determinant of recurrence, even during second-line rhythm-control interventions following initial CBA.

Despite technological advances in ablation methods over the past two decades, AAD options have remained largely unchanged. Flecainide and propafenone are still considered first-line agents for patients with AF without structural heart disease.^9,10^ Long-term use of these drugs has been associated with adverse events in 3.3%–7.7% of cases,^11–13^ with an average efficacy of approximately 52%. Data from the Euro Heart Survey on AF indicated usage rates of 17% for flecainide and 13% for propafenone in patients with paroxysmal or persistent AF.^14^ The relatively low preference for class IC agents may stem from the Cardiac Arrhythmia Suppression Trial (CAST) (1991), which reported increased mortality with these drugs in patients with ischemic heart disease.^15^ Therefore, screening for underlying structural or ischemic heart disease is essential before initiating class IC therapy. These agents should be avoided in patients with coronary artery disease, heart failure, or hypertensive LV hypertrophy.^1^

A meta-analysis of 60 studies reported short- and long-term efficacy of flecainide to be 65% and 45%, respectively.^16^ The mid-term efficacy of flecainide in our study was 65%, consistent with this meta-analysis. The long-term efficacy of both flecainide and propafenone has been reported to be approximately 52%.^17^ In our study, the combined 1-year sinus rhythm maintenance rate with these two drugs was 45%, aligning with previous literature. Importantly, our study is the first to demonstrate that flecainide is more effective than propafenone in maintaining sinus rhythm in patients with recurrent AF after CBA. The superior efficacy of flecainide in this population may be attributed to several factors: prior CBA with successful PV isolation, alterations in autonomic nervous system regulation (both sympathetic and parasympathetic), and changes in atrial myocardial cell depolarization properties. As is well known, CBA is an effective treatment for paroxysmal AF, comparable to RFA.^18,19^ In recurrent cases following cryoablation, three-dimensional (3D) electroanatomic mapping has shown that CBA creates long and durable lesions in the left atrium.^20^ In our study, as in other reports, 3D mapping in recurrent CBA cases frequently revealed reconnection in only a single PV, typically limited to a small segment of its circumferential lesion set. We hypothesize that the greater efficacy of flecainide therapy in this patient group may be attributed to the substrate modification achieved by CBA, resulting in AF triggers arising from a more localized region.

Flecainide is known to potentially induce atrial flutter with rapid ventricular response and therefore should not be administered as monotherapy. A β-blocker co-administration is strongly recommended.^1^ The combination of flecainide and metoprolol has been shown to exert a synergistic effect in paroxysmal AF by enhancing inhibition of Nav1.5 sodium channels, thereby reducing recurrence.^9,21^ In our clinic, all patients receiving flecainide were co-treated with metoprolol, which may explain the observed enhancement in efficacy within the flecainide group.

### Limitations

This study has several important limitations that should be acknowledged. First, it was a retrospective, single-center study conducted with a limited number of patients. A prospective, multicenter study with a larger patient population would provide more robust and generalizable results. Second, both flecainide and propafenone are associated with various cardiac and non-cardiac side effects,^9^ which may lead to treatment discontinuation in a proportion of patients. Due to the retrospective design of our study, we excluded patients who discontinued the medication within 1 year of follow-up, particularly those who stopped treatment due to adverse effects. This approach allowed us to evaluate the direct therapeutic efficacy of the drugs without the confounding influence of early discontinuation. It is worth noting that previous studies have reported a higher discontinuation rate for flecainide due to side effects compared to propafenone.^17^ This factor may partially explain the greater efficacy of flecainide observed in our study. Third, we only evaluated class IC AADs, specifically flecainide and propafenone, in this study. However, other AAD classes are commonly used in patients with paroxysmal AF and concomitant structural heart disease. In addition, patients receiving other class IC agents, dronedarone, amiodarone, or sotalol were not included in the study. Including such patient populations and comparing multiple AAD classes might have provided a broader perspective. Fourth, only patients with paroxysmal AF were included in the study. Inclusion of persistent AF patients could have added further insight into the relative efficacy of the treatment strategies across different AF subtypes. Fifth, the lower efficacy of propafenone therapy observed in our study may be attributed to the relatively low dosage used (150 mg three times daily) and the fact that higher doses (up to the maximum daily dose of 600–900 mg) were not administered. Sixth, according to the 2024 joint consensus statement on AF ablation, it is recommended that patients undergo at least 24 h of continuous Holter-type monitoring every 3 months during the first year following ablation.^18^ In symptomatic patients, longer-duration recordings with 7-day or 14-day continuous monitoring are preferable.^18^ In our study, we performed 72-h ambulatory rhythm Holter monitoring at 3-month intervals and repeated the same evaluation in symptomatic patients. Finally, this study exclusively involved patients who had undergone CBA and subsequently developed AF recurrence. Other ablation techniques, such as RFA or pulsed field ablation (PFA), are also used in clinical practice. It has been demonstrated that AF ablation using PFA can be performed with efficacy comparable to that of cryoballoon and RFA, while maintaining a lower complication rate.^18,22^ In the future, PFA integrated with 3D mapping systems may serve as an effective treatment option, particularly for patients with recurrent AF. The post-ablation recurrence profile and the effectiveness of class IC agents may differ in these patient groups, limiting the generalizability of our findings to those ablation modalities.

## Conclusion

Based on the findings of our study, flecainide therapy may be considered an effective treatment option with an acceptable success rate in patients with recurrent AF following CBA. In patients who initially received flecainide as a first-line treatment but later experienced AF recurrence, RFA may be considered as the subsequent rhythm-control strategy. Furthermore, among the class IC AADs, flecainide was found to be more effective than propafenone in maintaining sinus rhythm in patients with recurrent AF after CBA. These findings support the current guideline-based approach that recommends initiating class IC agents in patients with paroxysmal AF without structural heart disease and considering ablation therapy for those who are unresponsive to drug therapy. However, to validate and generalize these observations, prospective, multicenter studies with larger patient populations are warranted.
